# Effects of nocturnal celestial illumination on high-flying migrant insects

**DOI:** 10.1098/rstb.2023.0115

**Published:** 2024-06-24

**Authors:** Boya Gao, Gao Hu, Jason W. Chapman

**Affiliations:** ^1^ Department of Entomology, Nanjing Agricultural University, Nanjing 210095, People's Republic of China; ^2^ Centre of Ecology and Conservation, University of Exeter, Penryn, Cornwall TR10 9FE, UK; ^3^ Environment and Sustainability Institute, University of Exeter, Penryn, Cornwall TR10 9FE, UK

**Keywords:** nocturnal insect migrants, moon illumination, flight activity, orientation

## Abstract

Radar networks hold great promise for monitoring population trends of migrating insects. However, it is important to elucidate the nature of responses to environmental cues. We use data from a mini-network of vertical-looking entomological radars in the southern UK to investigate changes in nightly abundance, flight altitude and behaviour of insect migrants, in relation to meteorological and celestial conditions. Abundance of migrants showed positive relationships with air temperature, indicating that this is the single most important variable influencing the decision to initiate migration. In addition, there was a small but significant effect of moonlight illumination, with more insects migrating on full moon nights. While the effect of nocturnal illumination levels on abundance was relatively minor, there was a stronger effect on the insects' ability to orientate close to downwind: flight headings were more tightly clustered on nights when the moon was bright and when cloud cover was sparse. This indicates that nocturnal illumination is important for the navigational mechanisms used by nocturnal insect migrants. Further, our results clearly show that environmental conditions such as air temperature and light levels must be considered if long-term radar datasets are to be used to assess changing population trends of migrants.

This article is part of the theme issue ‘Towards a toolkit for global insect biodiversity monitoring’.

## 1. Introduction

Insect populations are changing in rapid and dramatic ways worldwide. Many recent studies have presented evidence of pronounced long-term regional declines in overall abundance, biomass and diversity, across a wide range of taxa [1–3]. These alarming results have led to widespread concern and debate about the role of, e.g. habitat loss and fragmentation, urbanization, pollution and climate change, in driving the potential crisis in insect communities. By contrast, some studies have demonstrated that population trends are more complex and nuanced, with a mix of decreasing, stable and increasing abundance and distribution trends, often co-occurring in the same insect communities [3–7].

Ecological traits are an important driver of variability in the slope and direction of abundance and distribution trends, with positive trends often associated with traits such as high mobility, wide host ranges, ecological generalization, broad thermal tolerance and large geographical ranges [5–9]. This combination of ecological attributes is generally considered to be part of the insect ‘migratory syndrome’, a set of behavioural, morphological, physiological and genetic traits common to migrant species [10,11]. As such, one may predict that migratory insects will tend to have more positive abundance and/or distribution trends than non-migratory species.

Given that many insect migrants are serious crop pests [12] and/or important disease vectors [13,14], while others provide important ecosystem services such as pollination [15,16] and biological control [17,18], it is increasingly important to monitor movement patterns, phenology and abundance of migratory insects. This is an extremely challenging endeavour, however, for a variety of reasons, two of which we briefly highlight. First, most species migrate by flight hundreds of metres above the ground [11], which means they cannot be monitored by traditional methods while engaged in migratory flight. Second, in many insect orders, and in many regions, knowledge of which species among local insect communities are migratory is largely incomplete [19], and thus it is not possible to monitor suites of migrant species when they are not migrating. One solution to these problems is to ignore individual species identity and use remote sensing data on abundance of migrating insects in the atmosphere, collected either purposefully by specialized biological radars [20,21] or as a side-product by networks of weather surveillance radars [22], to monitor spatio-temporal trends in aerial abundance. Radar data hold great promise for general monitoring of aerial insect migrants [23], but to correctly interpret any trends that may be apparent in long time-series, it is essential to fully understand the influence of environmental variables on the activity patterns, intensity and behaviour of airborne migrants.

Local weather conditions are one of the most important cues involved in the initiation and termination of animal migrations. In airborne insect migrants, temperature and windspeed at ground level have opposing effects on take-off decisions, with warmer temperatures and lighter windspeeds promoting intense migrations of day-flying insects [24]. By contrast, the influence of natural night-time illumination levels on high-altitude nocturnal insect migration has been less studied. Ambient illumination available to high-flying migrants from natural sources will vary substantially from night to night, even at the same location. This is because the amount of celestial illumination reaching migrants in flight depends upon: (i) the percentage of the moon that is illuminated; (ii) the moon's elevation above the horizon (i.e. whether the moon has risen high enough during the flight period to be visible to the migrants); and (iii) the degree of cloud cover above the migrants, as heavy cloud will prevent moonlight (and starlight) from reaching the migrants.

The degree of natural nocturnal illumination that reaches the migrants may be expected to influence the decision rules, flight activity and flight behaviour of high-flying insects for a variety of reasons, though there is very little research on this topic in actively migrating insects. Moon phase is known to synchronize timing of hatching and reproduction in a variety of animal taxa, most notably in marine, intertidal and freshwater species [25], including some insects with aquatic stages, e.g. Ephemeroptera, Chironomidae and Culicidae [26,27]. Recently, moon phase has been implicated in the activity patterns of some night-active birds, including synchronization of the timing of nocturnal migration in European nightjars (*Caprimulgus europeaus*) [28], and driving nocturnal vertical flight dynamics in American black swifts (*Cypseloides niger*), common swifts (*Apus apus*) and pallid swifts (*Apus pallidus*), which stay continuously airborne for many months [29,30]. There is comparatively little research on the effect of moon phase on nocturnal insect flight activity, and what does exist is concerned with low-level (i.e. non-migratory) flight sampled by light traps and suction traps within a few metres of the ground [27,31–34], so it is not currently known if lunar phase has any synchronizing effect on high-altitude insect migration. However, given that most nocturnal insect migrants are relatively short-lived as adults, with a brief window of opportunity (often just several nights) in which to undertake migratory flights [11], the pressure that migrants are under to complete the migratory phase of their life cycle seems incompatible with the lengthy delays required to synchronize with phases of the moon. We therefore assume it is unlikely that lunar phase *per se* will control large-scale migratory dynamics in populations of nocturnal high-flying insects.

However, there may be behavioural effects of the level of nocturnal illumination provided by the moon (rather than the specific lunar phase) on the decision to initiate migration on any night, if other factors known to be important are also conducive to take-off. For example, if migrants (for whatever combination of reasons, see below) have a preference for flight on nights of either low or high illumination, then the number of prospective migrants initiating flight may be influenced by the percentage of the moon that is illuminated, and the visibility of the moon (affected by whether the moon is above or below the horizon, and also the degree of cloud cover). Insects may prefer to migrate on moonlit nights to reduce the risk of predation by insectivorous bats, which prefer to forage on darker nights to reduce their own predation risk [25]. Conversely, nocturnally active insectivorous birds such as nightjars (which lack echolocation) forage more on moonlit nights [25,28], and thus insect migrants may prefer darker nights if bird predation is more of a risk than bat predation. Alternatively, they may reduce predation risk by increasing their flight altitude above the typical foraging range of both groups of predators on either moonlit [29] or moonless nights, again dependent on whether bird or bat predation is the more significant threat.

Insect migrants may also select nights with high illumination to facilitate visually guided navigation [35]. Published evidence suggests it may be challenging for nocturnal insect migrants to use a stellar or lunar compass to select a favourable compass direction [36,37]. However, they might be able to make use of increased light levels to improve aspects of their navigational capability, such as the ability to determine the direction of high-altitude winds. Radar studies have indicated that high-flying nocturnal insects can identify the downwind direction of the airstream in which they are flying by physically sensing turbulence cues associated with the flow [38–40], which would not rely on light levels. An alternative mechanism for sensing the flow direction is visual assessment of wind-induced drift with respect to the ground, by assessing the direction of the visual flow of ground features over the ventral surface of the eye. Nocturnal insects have exceptional night-vision under even very low light levels [41,42], and so this may be a plausible mechanism for detecting wind direction (and subsequently orientating with respect to the flow) under all illumination conditions that migrants would experience. However, notwithstanding the impressive visual acuity of nocturnal insects, a visual wind-detection mechanism would presumably be easier under higher illumination. Nocturnal insect migrants may thus prefer to fly under conditions of higher illumination for navigational purposes, and/or might be expected to be more proficient at identifying the wind direction at altitude when the night sky is brighter.

In this study, we use data collected by vertical-looking radars (VLRs) designed to monitor high-altitude insect migration [43] from above the southern UK to investigate the relationship between nocturnal insect flight activity and natural night-time illumination levels. Specifically, we test the effect of a range of celestial and weather variables on the numbers of insects migrating every night, their height of flight, and their ability to orientate close to the downwind direction. We predict that moonlight illumination levels and cloud cover may influence nightly migration intensity (but this will likely be a minor effect as many other factors such as temperature and wind conditions will also affect take-off decisions), but we do not have an *a priori* expectation about the direction of any trend as moonlight may encourage or discourage flight activity for the reasons discussed above. Additionally, we investigate any response that may occur in flight altitude in relation to illumination levels. We also predict that night-time illumination will have a positive effect on orientation performance with respect to the downwind direction.

## 2. Material and methods

### (a) Radar operating procedures

**Figure 1.**
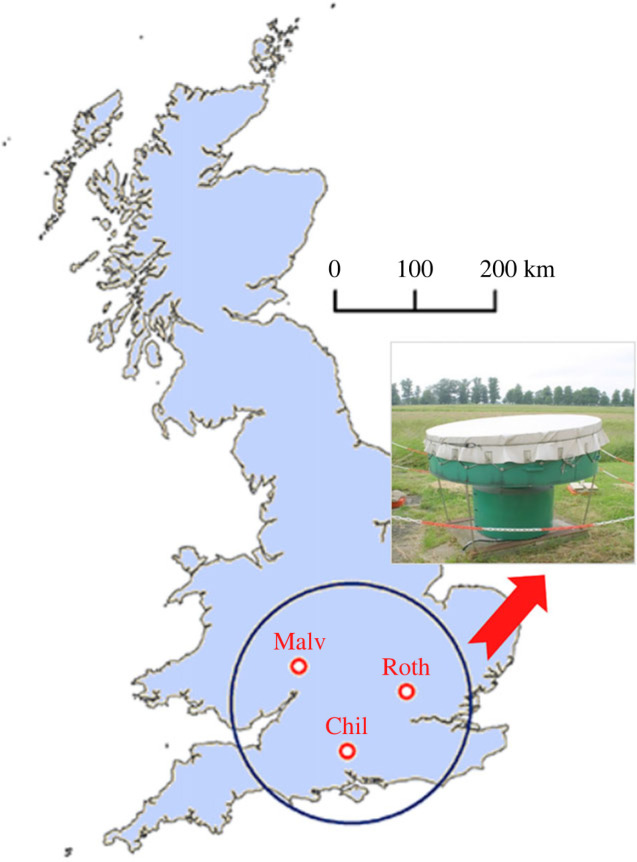
Nocturnal high-flying insect migrants were studied above a 70 000 km^2^ region of southern Britain (black circle) using data from vertical-looking radars (VLRs) at three locations (Rothamsted, Malvern and Chilbolton; red circles).

Insect abundance and flight behaviour data were collected by two vertical-looking insect radars (VLRs), located at three sites in southern England between 2000 and 2009. One VLR was located at Rothamsted Research, Harpenden, Hertfordshire (lat. 51°48′32″ N, long. 0°21′27″ W) for the whole 10-year period. The other VLR was at Malvern, Worcestershire (lat. 52°06′04″ N, long. 2°18′38″ W) from 2000 to 2003, and then at Chilbolton, Hampshire (lat. 51°8′40″ N, long. 1°26′13″ W) from 2004 to 2009 (figure 1). The VLR technology and operating procedure have been described in detail elsewhere [43]; however, we provide a summary below. The VLRs emit a vertically pointing, linear-polarized, narrow-angle conical scan. Insect targets are detected simultaneously from 15 height bands (each 45 m deep and separated by narrow non-sampling regions) between 150 and 1200 m directly above the radar during 5 min sampling periods, repeated every 15 min. This height range ensured that the targets analysed were high-flying insects; in other words, they were migrating. Signals from individual insects that transit the beam are returned and analysed, and include information on the time of transit, flight altitude, body mass and shape, displacement speed and direction, and flight heading for all resolved individuals. Hence, cumulative measures of the intensity of flight activity, e.g. aerial densities and flux rates, can also be calculated. To assess the effects of nocturnal illumination on migration intensity and flight behaviour of the nocturnal migratory insect fauna, we restricted our analyses of radar data to those collected from 1 h before sunset until midnight during major migration periods, which we have previously shown to be May to June (hereafter, the spring migration period) and August to September (hereafter, the autumn migration period) [20,44].

### (b) Acquisition and processing of meteorological and celestial data

We obtained all the meteorological and celestial data for our analyses from the Integrated Surface Database (ISD) of the National Oceanic and Atmosphere Administration (NOAA; https://www.ncei.noaa.gov), which provided weighted averages from the three nearest observation stations to our radar location that had data on each night. We used meteorological and celestial data from the spring and autumn migration periods from 2000 to 2009, thus aligning with the radar data. We used the following variables in our analyses. (a) Percentage moon illumination (i.e. the percentage of the lunar surface that is visibly illuminated, with 100% indicating a full moon and 0% indicating a new moon). (b) Moon rise time; if the moon did not rise above the horizon during our analysis period (1 h before sunset until midnight), in some of our models (see below for further details) we set the moon illumination value to 0% whatever the actual value, as it would not be visible to night-flying insects on that evening. (c) Surface-level temperature (1.5 m above the ground) at each radar site at sunset on each migration night. (d) Surface-level wind speed (10 m above the ground) of each migration night. (e) Percentage cloud cover (with 100% representing completely overcast, and 0% representing a completely clear sky) of each hour from 1 h after sunset to midnight for each migration night at each site.

These variables were used to analyse the impact of night-time illumination levels (i.e. the amount of moonlight available, and the amount of cloud present—which will affect the ability of insects to detect the moonlight that would otherwise be visible) on the number of insects migrating, their orientation capabilities, and their height of flight. The effect of percentage moon illumination on the migration variables was investigated in several ways. Firstly, we examined the distribution of the number of nights with each true moon illumination value, and then how this pattern changed when occasions when the moon did not rise above the horizon before midnight were assigned a 0% value. This led to a situation where there were no datapoints for moon illumination values below about 20%, but a very large number of datapoints with 0% illumination (electronic supplementary material, figure S1), presumably because lunar orbital patterns resulted in thin crescent moons not rising above the horizon before midnight during our spring and autumn seasonal periods. In one set of models, we examined the effect of the true moon illumination values (i.e. where we did not take moon rise time into consideration) on insect abundance and orientation performance; however, we felt that this did not give a true reflection of the effect of moonlight as on many of the occasions when moon illumination had a score above zero the moon was invisible below the horizon. We therefore ran another set of models where all occasions when the moon did not rise above the horizon were assigned a value of 0% illumination. However, we felt that the inclusion of a very large number of datapoints with a 0% illumination score might adversely impact our model outputs, for the following reasons. Firstly, the abundance of zero values may skew parameter estimation in the model distribution. Secondly, zero inflation may affect the model's fit, particularly around zero counts. Thirdly, convergence issues may arise during model estimation, especially if the optimization algorithm struggles to converge owing to the abundance of zero values. Lastly, negative binomial generalized linear models (GLMs) are designed to handle over-dispersion, but excessive zeros may exacerbate this issue. Thus, in a final set of models, all migration occasions with 0% moon illumination values (either true or assigned) were excluded from the analyses; while we compare the outputs of all three sets of models in the supplementary figures and tables, we present only the results from this final set of models (i.e. those models where zeros were excluded) in the main manuscript. For similar reasons, all occasions with zero insects were also excluded from the models (i.e. there had to be at least one insect flying for the occasion to be included in the analysis of abundance). In addition to moonlight and cloud cover, we also analysed the effect of surface-level meteorological data (temperature and wind speed), which are known to impact insect migration intensity.

### (c) Statistical analysis

Analyses were carried out separately for two categories of insect body size: medium (10–70 mg) and large (70–500 mg) insects, as previous studies have shown that migratory flight behaviour can differ substantially between these two size categories [20,40]. The presence of outliers, auto-correlation and collinearity in the meteorological and celestial factors were examined before we did further analyses. No collinearity was found between these variables, except there was a negative relationship between temperature and cloud cover (spring: correlation coefficient = −0.297; autumn: correlation coefficient = −0.335), but this did not influence the quality of our models. Our first analysis investigated the effect of the meteorological and celestial factors on the total abundance of individual insects in each size category that were detected above each radar site on every night during the spring and autumn migration periods of the 10 years of our study (from 2000 to 2009 inclusive). To model the abundance of insect migrants, a negative binomial generalized linear model (GLM was applied, after the initial Poisson GLMs indicated over-dispersion for both large and medium-sized insects. Auto-correlation and over-dispersion in the Pearson residuals of the fitted model were checked to justify the use of the negative binomial GLM.

To investigate how nocturnal illumination levels impact the orientation capability of nocturnal migratory insects, we investigated relationships between patterns of insect headings on each night, and two factors that will influence light levels, i.e. moon illumination percentage and cloud cover percentage. The VLRs recorded the body alignment of every individual insect, which represents the flight heading of the insect, albeit with a 180° ambiguity as it is not possible to distinguish the head-end from the tail-end by means of the radar signal alone. However, previous studies have demonstrated that, of the two possible values of the body alignment, the true insect heading is highly likely to be the one that is closest to the insect's direction of movement relative to the ground (i.e. its displacement direction or track) as this will be largely determined by the wind, and high-flying insects tend to orientate close to the downwind direction [20,40,44,45]. In the first step of this analysis, we therefore assumed that the body alignment value that was closest to the track direction was the true heading of each individual insect recorded by the VLRs.

Next, we restricted our analyses of insect orientation to migration occasions (i.e. a date/site/size combination) with more than ten insects detected, to produce meaningful circular statistical parameters for the group. Then we used the Rayleigh test [46] to produce the following parameters for each migration occasion: (i) the mean heading of the insects in each date/site/size combination; (ii) the mean vector length *r* (a measure of uniformity of the clustering of the headings around the mean, ranging from 0 to 1, with higher values indicating tighter clustering around the mean) for each migration occasion; and (iii) the probability that the distributions of headings differed from a uniform distribution (a *p*-value less than 0.05 indicates that the distribution of headings is significantly unimodal, and hence the individuals in that migration occasion showed a significant degree of common orientation of their headings). In the next stage, we selected migration occasions with significant common orientation (*p* < 0.05), and then investigated the effect of moon illumination and cloud cover on the size of the *r*-value associated with the distribution of individual headings on each occasion. We interpret higher *r*-values, indicating tighter group orientation close to the downwind direction, as indicative of improved orientation ‘performance’. We further assume that, if nocturnal illumination is positively associated with the ability to detect the wind and orientate accordingly, then *r*-values will be higher under the brighter conditions present when the percentage moon illumination is high and/or cloud cover percentage is low. Subsequently, for modelling the effect of these two variables on the orientation *r*-values, we used GLMs after checking that auto-correlation values of the variables were suitable for their use. Finally, the number of individuals on each migration occasion was used to weight the model when we applied the GLMs, to account for the reasonably large differences in the numbers of individuals from night to night [45]. Mean flight altitude of the insects on each night/site/size combination was also modelled in the same way, i.e. a GLM weighted by the number of insects on the migration occasion. As described in the §2b above, all moon illumination values of 0% (whether true or assigned values) were removed from the models investigating the effect of moonlight on abundance, flight altitude and orientation performance. To further check if moonlight illumination had any effect on abundance and orientation performance, we carried out pairwise comparisons of datapoints with 0% moon illumination against datapoints with moon illumination values greater than 0% (using a *t*-test where data were normally distributed, and a Wilcoxon test where they were not). All statistical analyses were carried out in the R software environment (v. 3.6.3).

## 3. Results

### (a) Migrant abundance

**Figure 2.**
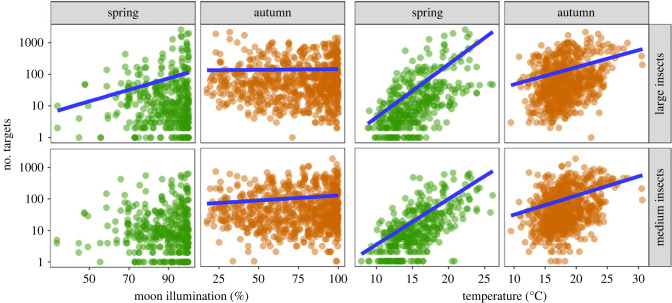
Abundance of spring and autumn insect migrants in each size class plotted against the two most important variables: temperature and percentage moon illumination. Insect abundance is plotted on a logarithmic scale. Blue regression lines are shown only if the variable in question was significant in the negative binomial generalized linear model (GLM), although the lines plotted are from the pairwise comparison rather than the GLM and therefore vary slightly from the model outputs. Associated statistics are presented in electronic supplementary material, table S1, and outputs of alternative analyses of the effect of moon illumination on abundance are presented in electronic supplementary material, figures S2 and S3; relationships with less important variables (wind speed and cloud cover) are shown in electronic supplementary material, figure S4 and table S1.

Migrant density varied by three orders of magnitude during migration occasions, from a single insect to more than 1000 insects during each date/site/size combination (figure 2; electronic supplementary material, figure S2). Temperature at take-off was the most important variable determining the abundance of migrants aloft, being highly significant (*p* < 0.0001 in all cases; figure 2 and electronic supplementary material, table S1). All relationships were positive, but were considerably steeper in spring than in autumn (large insects: spring *β* = 0.378, autumn *β* = 0.145; medium insects: spring *β* = 0.333, autumn *β* = 0.137). The other variable that had a relatively consistent, albeit much weaker, effect on abundance was percentage moon illumination. These relationships were again positive in all cases. In our final models (figure 2; electronic supplementary material, table S1), the effect was significant in both seasons in large insects (spring: *β* = 0.018, *p* = 0.0004; autumn: *β* = 0.001, *p* = 0.0286), but only during autumn in medium insects (*β* = 0.009, *p* < 0.0001). Similar patterns were observed in the models where the true moon illumination value was used irrespective of whether it rose above the horizon (electronic supplementary material, figure S2*a*), and in the models where zero values were included (electronic supplementary material, figure S2*b*). Further, pairwise comparisons of insect abundance when moon illumination was scored as 0% versus illumination greater than 0% also indicated that more insects migrated when the moon was visible (electronic supplementary material, figure S3). Our results therefore indicate that moon illumination had a consistent, albeit weak, positive effect on insect abundance. Cloud cover and wind speed had less clear and inconsistent relationships with abundance (electronic supplementary material, figure S4 and table S1), and thus we did not consider them of importance in determining insect abundance aloft.

### (b) Flight altitude

**Figure 3.**
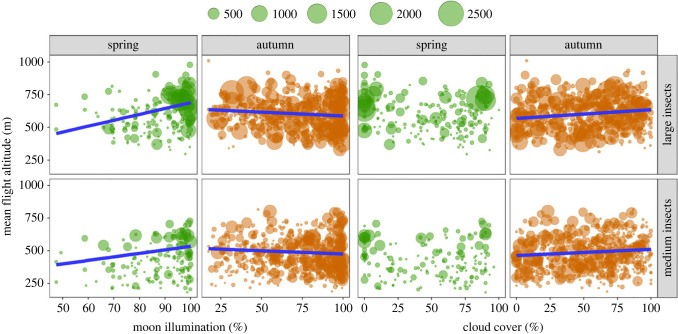
Flight altitude of spring and autumn insect migrants in each size class plotted against percentage moon illumination and percentage cloud cover. The number of insects detected by the radars on each nightly sample is indicated by the size of the green and orange circles and the key at the top of the panels. Blue regression lines are shown only if the variable in question was significant in the generalized linear model (GLM), although the lines plotted are from the pairwise comparison rather than the GLM and therefore vary slightly from the model outputs. Associated statistics are presented in electronic supplementary material, table S2.

Relationships between nocturnal illumination levels and flight altitude were less clear than the analyses of abundance and orientation performance, as the results were somewhat contradictory with respect to season. In both size categories, flight altitudes tended to be higher under brighter conditions during spring migrations as there were positive associations with moon illumination (large: *β* = 4.504, *p* < 0.0001; medium: *β* = 2.698, *p* = 0.0030; figure 3 and electronic supplementary material, table S2). By contrast, flight altitudes were higher during darker conditions in autumn, as there were negative associations with moon illumination (large: *β* = −0.583, *p* = 0.0044; medium: *β* = −0.493, *p* = 0.0360) and positive associations with cloud cover (large: *β* = 0.664, *p* < 0.0001; medium: *β* = 0.478, *p* = 0.0088; figure 3 and electronic supplementary material, table S2). Thus, the effect of nocturnal light levels on flight altitude is difficult to interpret and appears to vary with season.

### (c) Orientation performance

**Figure 4.**
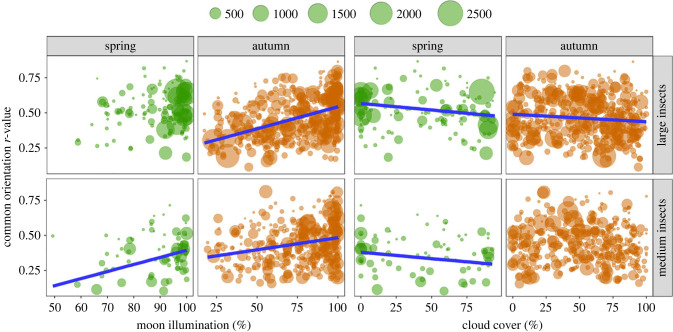
Orientation performance (*r*-value of the individual headings of all insects on a migration occasion) of spring and autumn insect migrants in each size class plotted against percentage moon illumination and percentage cloud cover. The number of insects detected by the radars on each nightly sample is indicated by the size of the green and orange circles and the key at the top of the panels. Blue regression lines are shown only if the variable in question was significant in the generalized linear model (GLM), although the lines plotted are from the pairwise comparison rather than the GLM and therefore vary slightly from the model outputs. Associated statistics are presented in electronic supplementary material, table S3, and outputs of alternative analyses of moonlight are presented in electronic supplementary material, figures S5 and S6.

Our measure of orientation ‘performance’, i.e. the magnitude of the *r­*-value resulting from circular analysis of the individual headings of insects on each migration occasion, varied from a high degree of spread (*r* < 0.2) to very tight clustering (*r* > 0.8) about the mean direction (figure 4). The GLM outputs of our final models (with no zero values in the moon illumination data) clearly indicated that orientation performance was significantly better on brighter nights (figure 4; electronic supplementary material, table S3). In large insects, orientation performance was positively associated with moon illumination in autumn (*β* = 0.003, *p* < 0.0001), and negatively associated with cloud cover in both seasons (spring: *β* = −0.001, *p* = 0.0010; autumn: *β* = −0.001, *p* = 0.0097). Medium insects showed a similar pattern, with orientation performance positively associated with moon illumination in both seasons (spring: *β* = 0.005, *p* < 0.0001; autumn: *β* = 0.002, *p* < 0.0001), and negatively associated with cloud cover in spring (*β* = −0.001, *p* = 0.0144). Similar patterns were observed in the models where the true moon illumination value was used irrespective of whether it rose above the horizon (electronic supplementary material, figure S5*a*), and in the models where zero values were included (electronic supplementary material, figure S5*b*); i.e. irrespective of the model used, the evidence consistently indicated that orientation performance was significantly improved when moon illumination was higher. Further, pairwise comparisons of orientation performance when moon illumination was scored as 0% versus illumination greater than 0% also indicated that insects were generally better at orientating close to downwind when the moon was visible (electronic supplementary material, figure S6). Our results therefore indicate that moon illumination had a consistently positive effect on insect orientation capabilities.

## 4. Discussion

In this study, we have used radar data to investigate the effect of natural illumination levels at night on the activity and flight behaviour of nocturnal insect migrants flying hundreds of metres above the ground. Previous work in this area has been restricted to observing these effects on or close to the ground, for example relating abundance of flying insects in ground-based suction and light traps to moon phase [27,31–34], or investigating the effect of celestial illumination on orientation responses of tethered insects [35]. Thus, to our knowledge, this is the first study to focus on these questions in migrant insects as they actively migrate high above the ground.

### (a) Migrant abundance

Previous studies of the influence of moon illumination on insect flight activity have provided a very mixed picture. Williams & Singh [31] showed that a mixture of flying nocturnal insects (primarily Diptera) sampled by ground-based suction traps in the UK were considerably more abundant around new moon than full moon, thus indicating that this community of insects preferred to fly under darker conditions. Brown & Taylor [32], suction trapping in East Africa, found contrasting results: total catches of all insects combined showed no response to lunar phase, but certain moths peaked at new moon and full moon, indicating relationships with lunar phase not directly linked to total illumination levels. Bowden & Gibbs [33] also suction trapping in East Africa, found that catches of pest Orthoptera and Lepidoptera tended to peak in the few days immediately following full moon, suggesting these species preferred brighter conditions. Finally, Danthanarayana's [34] results from suction trap samples of a range of insects in the UK provided the most complex picture; the data indicated a trimodal pattern of abundance within each lunar phase, with one peak coinciding with full moon, another with new moon, and yet another with intermediate illumination levels. Thus, no clear pattern emerges for relationships between flight activity and lunar illumination. Further, it is not clear if any of these ground-based studies are relevant to the current study, as the insects sampled may not have been migratory species or may not have been migrating at the time of capture.

Using radar detections of high-flying nocturnal insect migrants above the UK, we show that, of the four variables included in our models, surface-level temperature at the time of take-off is by far the most important driver of variation in abundance. This result for nocturnal insects is not surprising, as previous work has shown a positive relationship between warmer air temperatures and greater flight activity of day-flying migrants in the UK [24]. For the current study, the relationship is steeper in the spring (May and June) than in the autumn (August and September) migration period, presumably because temperatures are more limiting earlier in the year; migratory flight sometimes occurred when surface temperature at sunset was as low as 9–10°C in spring, but rarely below 12–13°C in autumn (figure 2).

In addition to temperature, percentage moonlight illumination also showed a consistent, albeit small, positive relationship with migrant abundance. Our models indicate that greater numbers of migrants fly on nights when the moon is close to full illumination. Although the moonlight effect was significant in three of the four size/season combinations, and had a similar (though not quite significant) trend in the fourth, we recognize that this effect was not very strong, and we encourage similar analyses to be carried out in other migrant insect communities. Should this apparent moonlight effect prove to be genuine, it is interesting to speculate on the reasons, as one might predict that migrants would prefer to fly on darker nights to reduce the risk of predation by visual predators (i.e. aerial insectivorous birds active at night). We suggest that this predation risk is relatively minor in temperate latitudes where nocturnally active insectivorous birds are relatively scarce, and thus any benefits accruing from flying on brighter nights will likely outweigh the potential costs. The benefit in question is likely that insect migrants have improved navigational capabilities under brighter conditions, as discussed below. Further work to assess the generality of the (slight) positive effect of moonlight on migrant flight activity that we observed, particularly in tropical regions where nocturnal insectivorous birds may be more frequent, is to be welcomed.

### (b) Flight altitude

The effect of nocturnal illumination on flight altitude of high-flying insects has not been directly studied before. Recent studies of flight activity in various species of swifts, tagged with multi-sensor data loggers, have shown that on their tropical wintering grounds these aerial insectivores remain continuously airborne day and night [29,30,47,48]. Night-time flight would provide opportunities for extended foraging if the swifts could see their potential prey, and so aerial foraging is presumably more likely to occur on moonlit nights; data from black swifts wintering in western Brazil suggests this is indeed the case, with flight activity patterns on full moon nights indicating active pursuit of prey [29]. The studies of tagged swifts also indicate that they fly higher on full moon nights, with pallid swifts and common swifts reaching about 1000 and 1500 m, respectively, above West and southern Africa [30], and American black swifts around 2000 m above Brazil [29]. These results led the authors of the swift studies to conclude that perhaps insect migrants also fly higher on full moon nights, thus driving the vertical flight dynamics of their aerial predators; however, there was no direct evidence that insects show this response. Our results provide partial support for this hypothesis, at least under certain circumstances, as we did find that during spring migrations there was a tendency for insects to fly higher when the moon was fully illuminated. The picture was complex, however, as during autumn migrations there was a tendency for insects to fly higher during darker conditions (when percentage moonlight illumination was low and percentage cloud cover was high). We therefore conclude that any relationships between insect flight altitude and night-time illumination remain unresolved, and we encourage more studies of this interesting question.

### (c) Orientation performance

Our measure of orientation ‘performance’ is the degree of angular dispersion of individual insects' flight headings around the group mean (represented by the *r*-value arising from the Rayleigh test) during migration occasions. We have previously demonstrated that high-flying insect migrants consistently take up flight headings relatively close to the downwind direction [20,40,44,45], and thus we assume that migration occasions with a smaller spread of headings around this mean (i.e. a higher *r*-value) indicate that insects are better able to detect the downwind direction under the conditions prevalent during that occasion. If visual assessment of displacement with respect to the ground is used to identify the downwind direction, then one would predict that this will be more easily performed under brighter night-time conditions. Our results strongly support this prediction, as headings were indeed more tightly clustered on brighter nights: there were positive relationships between *r*-values and moonlight illumination, but negative relationships between *r*-values and cloud cover, in three of the four season/size combinations. This suggests that vision is indeed important for the nocturnal navigation of high-flying insect migrants, as has been suggested from studies of compass orientation of migrant species under controlled conditions at ground level [35]. Physical sensing of the wind direction using micro-turbulence cues [38–40] is also an important mechanism, and is not dependent on ambient light levels, and so there is no indication that insects are incapable of this feat on dark nights. Rather, we conclude that on brighter nights, both sensory mechanisms are available to the migrants, and so group-level orientation performance is improved under these optimal conditions. This is presumably the principal reason for the (small) increase in migration activity that we observed on moonlit nights compared with darker ones.

## 5. Conclusion

Migratory insect communities are challenging to monitor for many reasons, not least the fact that we lack detailed knowledge of how many species are migrants [49]. Given the important roles that migrants play in shaping community dynamics, linking distant ecosystems, and transferring biological material including propagules and diseases [16,50,51], it is increasingly important that we find new ways to monitor their population trends and movement patterns. Networks of radars would seem to provide an ideal solution to this challenge, as they can provide long-term monitoring of population trends of aerial communities while simultaneously delivering important data for pest management and disease vector surveillance [23]. However, as the density of migrants in the atmosphere reflects not only population sizes on the ground, but also the suitability of conditions for flight, it is important to understand the influence of environmental conditions on flight activity. We have shown that for nocturnal insects migrating high above the UK, the most important variable is surface temperature at the time of take-off (dusk), and thus as air temperatures change throughout monitoring periods this factor will need to be accounted for when interpreting abundance trends. In addition, we show that nocturnal illumination levels from natural sources (moonlight and possibly starlight) can have small effects on aerial abundance, and stronger effects on flight behaviour. Our study was carried out in southern England, a relatively densely populated and urbanized part of the world where terrestrial light pollution levels are comparatively high [52], yet we still found an effect of natural illumination levels on orientation performance and flight activity; one may predict that these effects will be more pronounced in darker regions, and so further work on the role (if any) that anthropogenic light has on the behaviour and activity of nocturnal insect migrants is warranted. Further, any changes in cloud cover, and potentially in artificial light at night [52], will need to be considered when assessing population changes and activity patterns. Finally, our results strongly suggest that nocturnal insect migrants use celestial light sources to select and maintain wind-related flight headings, supplementing their physical wind-sensing mechanisms [38–40]. Further work to assess whether these results from the UK are generalizable to other aerial insect communities would be highly desirable.

## Data Availability

Data are available from the Dryad Digital repository: https://doi.org/10.5061/dryad.sn02v6xb5 [53]. Supplementary material is available online [54].
